# Mentalization deficits and entrapment moderate the association between complicated grief and suicide ideation among suicide-loss

**DOI:** 10.1192/j.eurpsy.2022.652

**Published:** 2022-09-01

**Authors:** L. Lev-Ari, Y. Levi-Belz

**Affiliations:** 1 Ruppin Academic Center, Clinical Psychology, Emek Hefer, Israel; 2 Ruppin Academic Center, Behavioral Sciences, hadera, Israel

**Keywords:** suicide-loss survivors, complicated grief, mentalization, suicide ideation

## Abstract

**Introduction:**

Suicide-loss survivors (SLSs) are recognized as an at-risk population for several psychiatric complications, including complicated grief (CG) and suicide ideation (SI). However, limited data are available concerning the contribution of CG to SI among suicide survivors. Moreover, knowledge about possible psychological processes which may increase SI levels following CG is rare.

**Objectives:**

In this study, we aim to examine the role of two important emotion regulation variables––mentalization deficits and entrapment––as possible moderators of the association between CG and SI in the aftermath of suicide loss.

**Methods:**

Participants were 152 suicide-loss survivors, aged 18-70, who completed questionnaires tapping CG, SI, mentalization deficits, and entrapment.

**Results:**

The findings revealed SI to have high and positive associations with CG, entrapment, and metallization deficits. Regression analysis showed mentalization deficits and entrapment contributing to SI beyond the contribution of CG. Notably, a significant interaction was found, indicating that CG and SI became more strongly associated at higher levels of mentalization deficits.

**Conclusions:**

The study’s findings highlight the critical link between complicated grief and suicide ideation among SLSs and the role of metallization deficits as a possible facilitator of this link. Practical implications relating to suicide risk among SLSs are discussed, as well as focused clinical recommendations. The importance of mentalization-based interventions for decreasing SI levels in the aftermath of suicide loss is highlighted.

**Disclosure:**

No significant relationships.

